# The Central Importance of Vaccines to Mitigate the Threat of Antibiotic-Resistant Bacterial Pathogens

**DOI:** 10.3390/vaccines13090893

**Published:** 2025-08-23

**Authors:** Jiaqi Amber Zhang, Victor Nizet

**Affiliations:** 1 The Bishop’s School, La Jolla, CA 92037, USA; amberzhangxie@gmail.com; 2 Division of Host–Microbe Systems and Therapeutics, Department of Pediatrics, University of California San Diego School of Medicine, La Jolla, CA 92093, USA; 3 Skaggs School of Pharmacy and Pharmaceutical Sciences, University of California San Diego, La Jolla, CA 92093, USA

**Keywords:** antimicrobial resistance, bacterial vaccines, multidrug-resistant pathogens, immunization, vaccine development

## Abstract

Antibiotics have dramatically reduced the burden of infectious diseases since their discovery, but the accelerating rise in antimicrobial resistance (AMR) now threatens these gains. AMR was responsible for nearly 5 million deaths in 2023 and continues to undermine the efficacy of existing treatments, particularly in low- and middle-income countries. While efforts to address AMR have focused heavily on antibiotic stewardship and new drug development, vaccines represent a powerful yet underutilized tool for prevention. By reducing the incidence of bacterial infections, vaccines lower antibiotic consumption, interrupt transmission of resistant strains, and minimize the selective pressures that drive resistance. Unlike antibiotics, vaccines offer long-lasting protection, rarely induce resistance, and confer indirect protection through herd immunity. This review examines the global burden and drivers of AMR, highlights the unique advantages of vaccines over antibiotics in mitigating AMR, and surveys the current development pipeline of vaccines targeting key multidrug-resistant bacterial pathogens.

## 1. Introduction

In the past century, antibiotics have significantly reduced the morbidity and mortality associated with infectious diseases. The antibiotic revolution began with Sir Alexander Fleming’s discovery of penicillin in 1928, with the 1950s to 1970s marking the golden era of antibiotic development [1]. However, antimicrobial resistance (AMR) has become increasingly problematic due to the rise in multidrug-resistant (MDR) bacteria. While AMR is a natural phenomenon, it has been accelerated by the overuse and misuse of antibiotics, which pressure pathogens to mutate into resistant forms more rapidly [2]. Misapplications include the use of antibiotics for viral infections—where they are ineffective—and empirical use without precise identification of bacterial infections, leading to unnecessary and inaccurate treatment [3]. Additionally, over-the-counter availability of antibiotics in many countries promotes unsupervised and excessive use [2]. Resistant bacteria are also spread through international travel and through the misuse of antibiotics in agricultural livestock under the guise of “therapeutic use”, resulting in consumption of excessive antibiotic residues [4]. This misuse contributes to environmental contamination via animal feces, further promoting AMR development [2].

AMR now severely limits the effectiveness of many antibiotics against previously treatable diseases, prompting the World Health Organization (WHO) to list it among the top ten global health threats in 2019 [5]. In 2019, a systematic review estimated the global death toll directly attributable to AMR at 1.27 million, a number that rises to 4.95 million when including all deaths associated with AMR [6], while other forecasts see annual AMR deaths reaching 10 million by 2050 [7]. The economic consequences of AMR are equally alarming: modeling studies suggest that uncontrolled AMR could cause a global GDP loss of 2–3.5% by 2050, with disproportionate effects on low- and middle-income countries (LMICs) [7].

Although multiple interventions have been implemented to reduce the burden of AMR—including antibiotic stewardship programs, improved sanitation and hygiene measures, public education campaigns, and funding mechanisms to incentivize new antibiotic development—these strategies alone are insufficient to curb the accelerating threat. Therefore, it is important to consider alternative therapeutic and preventative approaches beyond pharmaceutical antibiotics to combat AMR. This expanded toolkit includes several promising strategies, such as the development of monoclonal antibodies [8,9], bacteriophages [10], antivirulence therapies [11], probiotic or microbiome-based approaches [12], drug repurposing [13], and accelerated diagnostic tools [14,15].

Among these interventions, vaccination represents a critically important but underappreciated tool in the fight against AMR. While considerable attention has been devoted to preserving and extending the utility of antibiotics, less discussion has centered on preventing AMR infections altogether through immune-mediated protection. Vaccines, by reducing infection incidence, antibiotic consumption, severe disease progression, and the transmission of resistant pathogens, offer a sustainable and highly effective strategy to mitigate AMR. This review focuses on the role of vaccines in addressing AMR, examining their unique benefits, and evaluating the current status of vaccine development for key AMR bacterial species that we as authors have selected due to their clinical impact and high frequency of antibiotic treatment failures.

## 2. Advantages of Vaccines over Antibiotics in Confronting AMR

Patients with AMR infections experience longer hospital stays, more ICU admissions, more surgical interventions, and require protracted courses of broad-spectrum antibiotics—all of which perpetuate further AMR transmission within healthcare settings [16,17]. Preventing infections through vaccination interrupts this cycle at its origin, reducing severe disease that necessitates high-risk interventions and thereby serving as a primary strategy to limit hospital-associated AMR proliferation [14]. Importantly, targeted vaccination strategies can protect the most vulnerable populations—including neonates, the elderly, individuals with chronic lung diseases, immunocompromised patients, and those undergoing intensive medical interventions—who are at greatest risk for severe bacterial infections and the worst AMR outcomes [18]. Protecting these high-risk groups prevents not only individual morbidity and mortality but also blocks key nodes in the healthcare system where resistant infections are most likely to spread [19].

**Figure 1:**
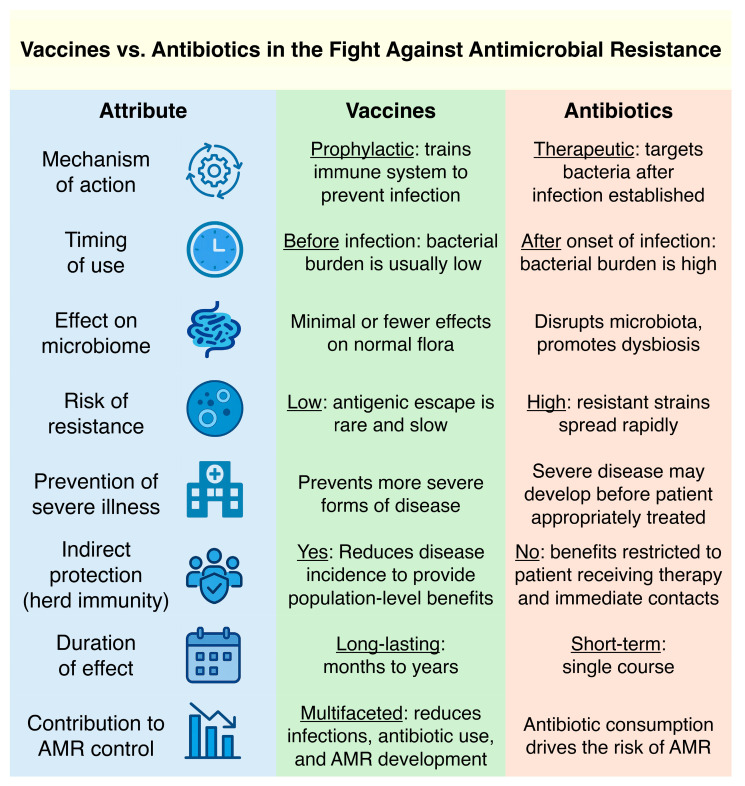
Comparative mechanisms and effects of vaccines and antibiotics in relation to AMR. This schematic table delineates key distinctions between vaccines and antibiotics across eight mechanistic and clinical attributes that shape AMR risk. Compared to antibiotics, vaccines act prophylactically at low pathogen burden, exert minimal disruption on commensal microbiota, and are less prone to inducing resistance due to the multifactorial nature of immune pressure. Additional advantages of vaccination include mitigation of severe disease progression, generation of herd immunity, and durable immune protection. In contrast, antibiotic therapy—while critical for established infections—applies strong selective pressure, often contributes to dysbiosis, and lacks both population-level benefits and durability of effect.

Vaccines and antibiotics operate via fundamentally different mechanisms, with profound implications for AMR dynamics (Figure 1). Antibiotics are administered reactively to treat established infections, often when bacterial populations are already large and heterogeneous. This creates a selective bottleneck favoring the survival and expansion of resistant mutants [20]. In contrast, vaccines are prophylactic interventions that train the immune system to recognize and eliminate pathogens early in infection, preventing disease establishment or significantly attenuating its severity [21]. Depending on the formulation, vaccination has the potential to reduce infections caused by both antibiotic-susceptible and -resistant bacteria. By preventing infections before they require antibiotic treatment, vaccines dramatically reduce antibiotic consumption and, in turn, the selective pressures that drive the evolution and dissemination of resistance [22].

Vaccines confer indirect protection through herd immunity, reducing pathogen circulation even among unvaccinated individuals and further limiting the opportunities for resistance selection [23]. Herd immunity also indirectly protects immunocompromised individuals who may not mount sufficient immune responses to a vaccine—a benefit antibiotics do not offer [14]. Another critical advantage of vaccines over antibiotics is their microbiome-sparing effect. Unlike antibiotics, which disrupt commensal microbial communities and promote the emergence of opportunistic and resistant organisms [24,25], vaccines are expected to exert targeted immunity with minimal impact on the microbiota [26]. Preserving microbial diversity is increasingly recognized as essential for maintaining immune homeostasis and preventing colonization by resistant pathogens [27].

Finally, vaccines provide long-lasting protection relative to antibiotics, often requiring only one or a few doses to achieve durable immunity over months or years. Critically, while bacterial pathogens can develop antibiotic resistance with alarming speed, vaccine resistance—through antigenic variation—is far less common and typically evolves much more slowly, owing to the multifactorial nature of immune pressure [28]. This difference in resistance dynamics may stem from vaccines’ ability to preemptively build immunity and their targeting of multiple microbial components, reducing the likelihood of pathogen mutation and the emergence of resistance [29,30]. Thus, vaccines offer a sustainable, population-wide approach to infectious disease prevention with minimal risk of inducing secondary resistance phenomena.

## 3. Lessons from Successful Vaccination Campaigns Against AMR

The above considerations are not merely theoretical—historical and contemporary vaccine programs provide real-world evidence of these principles in action. The introduction of *Haemophilus influenzae* type B (Hib) conjugate vaccines led to a dramatic global reduction in invasive Hib disease incidence, alongside a significant decrease in beta-lactamase-producing strains [31,32]. In the pre-vaccine era, Hib infections drove widespread empirical antibiotic use, often involving broad-spectrum agents. Following the introduction of the Hib vaccine, not only did disease rates plummet, but resistance trends reversed in many regions, validating the dual benefit of vaccination [30].

A similar pattern emerged with pneumococcal conjugate vaccines (PCVs). The introduction of PCV7 and its successor, PCV13, led to significant reductions in invasive pneumococcal disease (IPD) caused by vaccine-covered serotypes, many of which were multidrug-resistant. For instance, one study reported a 57% decline in IPD caused by penicillin-nonsusceptible strains following the introduction of PCV7 [33]. Similarly, another analysis observed a 74.1% decrease in dual macrolide-resistant IPD—strains that contain both *mef(E)/mel* and *erm(B)* genes—after the implementation of PCV13 [34]. Crucially, herd immunity extended protection to unvaccinated adults [35], amplifying reductions in antibiotic prescriptions and the prevalence of resistant strain at the population level.

Even viral vaccines have demonstrated indirect effects on AMR. Influenza vaccination reduces the incidence of secondary bacterial infections such as pneumococcal pneumonia and otitis media, thereby lowering antibiotic use and inappropriate prescribing. A systematic review and meta-analysis found that influenza vaccination significantly reduces antibiotic use, with a risk ratio (RR) of 0.63 (95% CI 0.51–0.79) for the proportion of people receiving antibiotics after vaccination [36]. For example, the implementation of a universal influenza immunization program in Ontario, Canada, was associated with a 64% reduction in influenza-associated antibiotic prescriptions compared to provinces with limited vaccine coverage [37]. Furthermore, by decreasing the incidence of viral syndromes that are frequently mismanaged with antibiotics, influenza vaccination contributes meaningfully to stewardship efforts [38].

## 4. Pathogen-Specific Vaccine Strategies to Combat AMR

**Figure 2:**
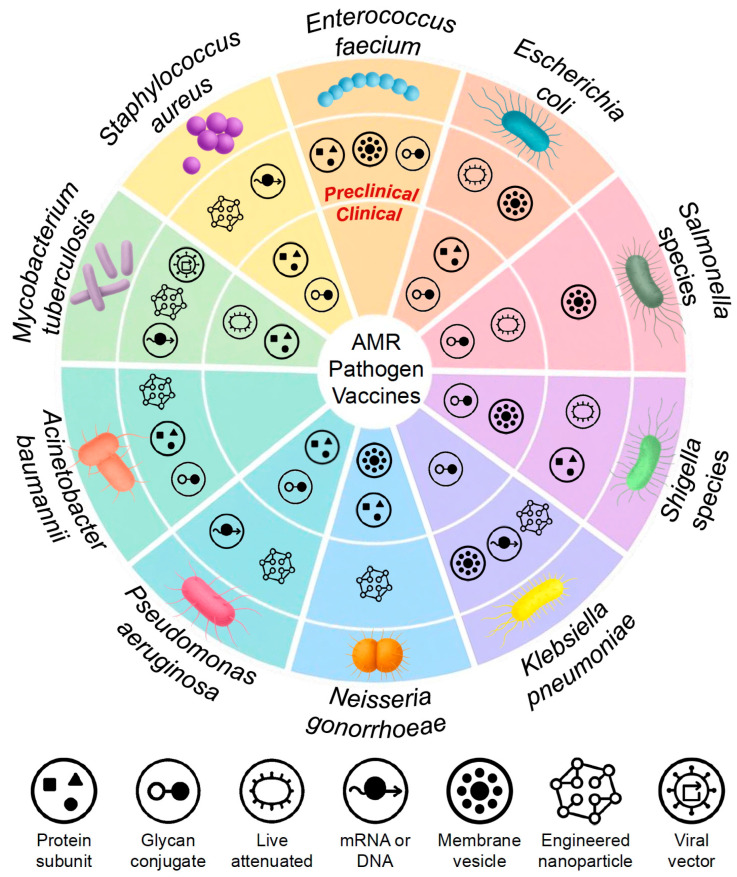
Landscape of vaccine development against leading AMR bacterial pathogens. This circular schematic summarizes vaccine candidates under preclinical (outer ring) and clinical (inner ring) investigation for 10 prioritized AMR bacterial pathogens. Each wedge corresponds to a pathogen, with icons representing the vaccine platform used: protein subunit, glycan conjugate, live attenuated, mRNA/DNA, membrane vesicle, engineered nanoparticle, or viral vector. The figure illustrates the diversity of vaccine technologies being applied and highlights areas with greater or more limited development progress.

Having established the rationale and overarching benefits of vaccination in addressing antimicrobial resistance, we now turn to a pathogen-specific examination. The following sections highlight the current status, scientific challenges, and recent advances in vaccine development for key bacterial species prioritized due to their clinical impact and resistance profiles. Figure 2 provides a comparative overview of vaccine candidates in preclinical and clinical development across these AMR pathogens, illustrating the diversity of platforms under investigation—including protein subunits, glycan conjugates, mRNA, nanoparticles, and outer-membrane vesicles.

### 4.1. Mycobacterium tuberculosis

Tuberculosis (TB), caused by *Mycobacterium tuberculosis* (Mtb), remains a leading cause of infectious disease mortality, with an estimated 10.6 million new cases and 1.6 million deaths globally in 2021 [39]. The burden is particularly high in Asia and Africa, which together account for over half of all cases [39]. The emergence of AMR in TB poses significant challenges. In 2021, approximately 450,000 cases were multidrug-resistant TB (MDR-TB), and 25,000 were extensively drug-resistant TB (XDR-TB) [40]. Treatment success rates are low—only about 54% for MDR-TB and 28% for XDR-TB—underscoring the urgent need for effective vaccines [41].

TB vaccine development presents unique and formidable challenges, rooted both in the biological complexity of Mtb and the limitations of current experimental systems. Mtb is a master of immune evasion, persisting within macrophages by inhibiting phagosome-lysosome fusion and subverting host signaling pathways to limit antigen presentation and immune activation [42,43]. Its ability to enter a latent, non-replicating state further complicates immune targeting and necessitates vaccines that can prevent both primary infection and reactivation. Despite decades of research, no validated immune correlate of protection exists for TB. While Th1-type responses—particularly IFN-γ production—are essential, they are insufficient alone to predict vaccine efficacy, posing a major barrier to rational design and early candidate selection [44,45]. Only recently have antibodies been recognized to play a functional role to Mtb immunity [46]. Compounding these issues is the lack of animal models that faithfully recapitulate human disease: mice fail to develop granulomas with human-like pathology, and non-human primate models, while more representative, are costly and limited in throughput [47,48].

Currently, the Bacille Calmette–Guérin (BCG) vaccine is the only licensed vaccine against Mtb. Introduced in 1921, BCG is derived from a live-attenuated strain of *Mycobacterium bovis,* a close relative of Mtb. It is widely administered in TB-endemic regions, typically at birth or during early childhood [49]. However, BCG’s utility is limited by several factors. Its live-attenuated nature makes it unsuitable for immunocompromised individuals, including those infected with HIV—one of the populations most vulnerable to TB [50]. Although BCG protects against certain forms of TB, particularly severe pediatric disease, its efficacy against pulmonary TB—the most common and transmissible form—varies significantly by geography. For example, the vaccine has demonstrated high efficacy in the United Kingdom but minimal protection in South India [51]. This variability may reflect environmental exposure to non-tuberculous mycobacteria, which are more prevalent near the equator and may interfere with BCG-induced immunity [51].

Global efforts have focused on developing next-generation TB vaccines with more consistent and robust efficacy, particularly in high-burden settings. Some promising candidates are now advancing through clinical trials. VPM1002, a recombinant BCG vaccine engineered to improve antigen presentation and immunogenicity, is undergoing phase 3 trials in India and sub-Saharan Africa [52,53]. A phase 2 study in South Africa demonstrated comparable safety and immunogenicity to BCG, with reduced local reactogenicity in neonates [53]. MTBVAC, the only candidate based on a live attenuated strain of Mtb itself (rather than *M. bovis*), has progressed to phase 3 trials evaluating efficacy in HIV-exposed and unexposed newborns in Africa [54,55]. Early clinical studies confirmed its safety and superior immunogenicity relative to BCG [56]. Another leading candidate, M72/AS01E—a protein subunit vaccine combining two Mtb antigens (Mtb32A and Mtb39A) with the AS01E adjuvant—demonstrated 54% protection against progression to active TB in adults with latent infection during a phase 2b trial across Kenya, South Africa, and Zambia [57,58]. This represented one of the most promising efficacy signals seen in decades of TB vaccine research. In parallel, strategies to boost or extend BCG-induced immunity are being explored. The quadrivalent subunit vaccine HEHR, formulated with Hsp90, ESAT-6, HspX, and RipA, and adjuvanted with CIA09A, showed enhanced protection in preclinical mouse models when used as a BCG booster, delivered either intramuscularly or subcutaneously [59]. This approach could improve BCG durability in high-burden populations.

A diverse array of preclinical TB vaccine candidates are being developed using innovative platforms attempting to elicit more robust and durable immunity. mRNA-based vaccines, modeled after successful COVID-19 platforms, have shown promise in mice—eliciting strong antigen-specific T cell responses and reducing bacterial burden when encoding TB antigens such as Ag85B and ESAT-6 [60]. Nanoparticle vaccines, including lipid–polymer hybrids and self-assembling protein nanoparticles, offer enhanced antigen delivery and immune activation; several have demonstrated improved protection and mucosal immunity in preclinical challenge models [61,62,63]. Viral vectors remain a mainstay among novel TB strategies: recombinant MVA (e.g., MVA85A) and ChAdOx1-based adenoviral vectors encoding TB antigens have shown immunogenicity in both animal models and early-phase trials, though efficacy remains to be validated in larger studies [64,65].

TB-endemic settings, however, will continue to pose clinical trial challenges for vaccine innovation. Most adults already have latent TB infection, so effective vaccines must either prevent disease progression or block reinfection—each requiring distinct immune responses. Co-infections such as HIV and comorbidities like diabetes further complicate immunogenicity and efficacy evaluation [66]. Together, these factors make TB vaccine development especially complex and resource-intensive, underscoring the need for novel platforms, surrogate markers, and integrated global strategies. While these candidates mark important milestones, TB vaccine development remains a long-term endeavor that must be integrated with complementary public health interventions—such as improved diagnostics, shortened treatment regimens, and transmission reduction strategies—to achieve global TB control.

### 4.2. Staphylococcus aureus

*Staphylococcus aureus*, including prevalent methicillin-resistant (MRSA) and occasional vancomycin-intermediate or -resistant (VISA, VRSA) strains, remains a leading cause of skin and soft tissue infections (SSTIs), bacteremia, sepsis, osteomyelitis, endocarditis, pneumonia, and toxic shock syndrome in both hospital and community settings. An effective *S. aureus* vaccine is urgently needed, but none to date have achieved clinical success.

One major challenge unique to the *S. aureus* is its abundant surface-expressed Protein A (SpA), which complicates natural and vaccine-induced adaptive immunity by (a) binding with high affinity to the Fc region of IgG, interfering with opsonophagocytosis [67], and (b) engaging the Fab region of variable heavy chain 3 (VH3) family B-cell receptors, inducing non-specific polyclonal B-cell activation that impairs the development of robust, durable, and antigen-specific humoral immune response [68]. Another challenge is that prior exposure to *S. aureus* impairs vaccine efficacy by inducing immune imprints that generate and recall non-neutralizing IgGs with altered glycosylation, blunting opsonophagocytosis [69]. Likewise, *S. aureus*-experienced CD4+ T cells secrete IL-10, suppressing IL-17A production and weakening vaccine-induced protection [70], hinting that special adjuvant formulations may be required for the pathogen.

A number of *S. aureus* vaccine candidates progressed to clinical efficacy trials over the years, but with disappointing results. V710 (Merck), targeting the iron-scavenging protein IsdB, was studied in patients undergoing cardiothoracic surgery, but its Phase 3 trial was terminated when it failed to reduce the rate of serious postoperative *S. aureus* infections and raised safety concerns [71]. StaphVAX (Nabi Biopharmaceuticals), a conjugate vaccine targeting capsular polysaccharides CP5 and CP8, advanced to Phase 3 trials in hemodialysis patients but found no reduction in *S. aureus* types 5 and 8 infections in the StaphVAX group vs. the placebo group [72]. SA4Ag (Pfizer), a multivalent vaccine containing CP5 and CP8 conjugates, clumping factor A (ClfA), and MntC, was well-tolerated and elicited substantial antibody responses but was not efficacious in preventing *S. aureus* infection site or bloodstream infections in patients undergoing elective open posterior spinal fusion surgeries [73].

Since the above *S. aureus* vaccine programs—designed to generate high titers of opsonic antibodies against surface antigens—failed in advanced trials, many have argued that targeting the pathogen’s pore-forming toxins and superantigens could be a more fruitful strategy than relying solely on antibody-mediated bacterial clearance [74]. For example, *S. aureus* pore-forming leukocidins LukED and HlgAB suppress antibody responses during bloodstream infection in mice, but vaccination with these toxoids prevented immune subversion, yielding neutralizing antibodies, effective Th1/Th17 responses, and protective immunity [75]. In another study, immunization with both alpha-toxin (Hla) and Panton–Valentine leukocidin (PVL) elicited broadly cross-neutralizing antibodies that protected against death in a rabbit model of MRSA USA300 necrotizing pneumonia [76]. In a first-in-human trial, immunization with a recombinant toxoid form of superantigen toxic shock syndrome toxin 1 (TSST-1) elicited antibodies that efficiently neutralized T cell hyperactivation ex vivo [77,78]; this candidate has now demonstrated safety and robust immunogenicity in phase 2 trials [79]. LBT-SA7 (LimmaTech, Switzerland), a five-component vaccine comprising seven *S. aureus* toxoids to prevent SSTIs, has received U.S. FDA fast-track approval to commence Phase 1 safety and immunogenicity studies (NCT06719219), and GSK likewise has a pentavalent candidate (SA-5Ag) in clinical trials for the same indication (NCT04420221). for Finally, one recent candidate combines secreted toxins and surface components: rFSAV (Olymvax) includes staphylococcal enterotoxin B (SEB), α-hemolysin (Hla), IsdB, MntC, and staphylococcal protein A (SpA), and has exhibited low reactogenicity and high immunogenicity in phase 1 studies and a phase 2 trial among patients undergoing elective orthopedic surgery [80,81].

NDV3-A (Novadigm) is an adjuvanted vaccine comprising the N-terminal domain of agglutinin-like sequence 3 protein (Als3p) from the cell wall of *Candida albicans* [82]. Originally developed for fungal infections, structural similarities between Als3p and the surface-anchored ClfA protein of *S. aureus* have allowed for its repurposing [83]. While NDV3-A demonstrated safety and immunogenicity in phase 1 trials [84], a subsequent phase 2 trial found that a single dose did not prevent nasal or oral acquisition of *S. aureus* among U.S. Army infantry trainees at high risk for colonization [84]. Whether further studies involving multi-dose regimens will be pursued remains unclear.

Preclinical studies have explored a novel approach in which nanoparticle cores cloaked in natural human red blood cell membranes to retain and present *S. aureus* staphylococcal alpha-toxin (Hla). The engineered particles efficiently stimulated germinal center formation, induced high anti-Hla titers, and protected mice against MRSA skin challenge [85]. In a first report of mRNA vaccine technology targeting *S. aureus,* immunization against SEB led to strong antibody responses and significantly reduced mortality and bacterial burdens in both blood and organs of challenged mice, outperforming protein-based controls [86].

Finally, targeting cellular immunity against *S. aureus* has emerged as a promising alternative strategy for vaccine development. Protective immunity to *S. aureus* relies not only on opsonic antibodies but also on robust T cell responses. In particular, IL-17–producing CD4^+^ T cells (Th17) are critical for neutrophil recruitment and abscess containment, as evidenced by murine models where IL-17 deficiency results in impaired clearance of *S. aureus* skin infections [87]. Likewise, Th1 cells producing IFN-γ enhance macrophage bactericidal function and contribute to systemic control of infection [88]. The importance of cellular immunity is underscored by the clinical susceptibility of patients with genetic defects in Th17 pathways, such as STAT3 mutations in hyper-IgE syndrome, who suffer from recurrent *S. aureus* infections [89]. Recent efforts have therefore sought to design vaccines that stimulate balanced humoral and cellular immunity. For example, co-administration of the 4C-Staph antigen with the MF59 adjuvant significantly enhanced both antibody titers and CD4^+^ T cell responses in preclinical models [90], while transient IL-10 blockade during vaccination has been shown to amplify Th1/Th17 polarization and improve protective efficacy [91]. Vaccination with S. aureus-derived extracellular vesicles (EVs) conferred protection in mice against lethal and sublethal airway challenge primarily through Th1-mediated cellular immunity, with protective effects transferable by T cells but not serum [92]. Collectively, these findings support the view that successful *S. aureus* vaccines will need to elicit coordinated cellular and humoral responses, overcoming the limitations of antibody-centric approaches [93].

### 4.3. Enterococcus spp.

*Enterococcus* is a major cause of nosocomial infections, including urinary tract infections (UTIs), bacteremia, and endocarditis, with *E. faecalis* and *E. faecium* being the most clinically significant species [94,95]. Its intrinsic and acquired antimicrobial resistance—particularly the emergence of vancomycin-resistant strains since the 1980s—poses a significant therapeutic challenge [94,95]. However, limited understanding of natural immunity to *Enterococcus* and the structural complexity of suitable antigen targets have constrained vaccine development to preclinical research [96].

One promising approach targets the fibrinogen-binding domain of the pilus tip adhesin EbpA. A vaccine utilizing EbpA or its amino-terminal domain effectively inhibited biofilm formation and protected mice against catheter-associated UTIs [97]. Another study demonstrated that vaccination with *E. faecium* outer-membrane vesicles (OMVs) elicited immune responses in rabbits and provided cross-protection against multiple strains, including vancomycin-resistant isolates, though further research is needed to evaluate its translational potential [98].

A third strategy has explored glycoconjugates to improve cross-strain coverage for *E. faecium* and *E. faecalis*. In this approach, the *E. faecalis* polysaccharide diheteroglycan was conjugated to either secreted antigen A (SagA) or peptidyl-prolyl cis-trans isomerase (PpiC)—two *E. faecium* proteins previously shown to elicit cross-protective and opsonic antibodies [99]. In a rabbit model, both conjugates induced immunoreactivity against diverse strains of *E. faecalis* and *E. faecium*, supporting their potential as broad-coverage vaccine candidates [99].

### 4.4. Escherichia coli

Extraintestinal pathogenic *Escherichia coli* (ExPEC) is the most prevalent Gram-negative bacterial pathogen in humans. It is responsible for most UTIs [100], a leading cause of adult bacteremia [101], and the second most common cause of neonatal sepsis and meningitis [102]. Rising multidrug resistance in invasive ExPEC strains presents a significant therapeutic challenge, contributing to increased hospitalizations, higher mortality rates, and escalating healthcare costs [103].

Only two ExPEC vaccines have advanced to clinical development. ExPEC9V, a bioconjugate polysaccharide vaccine developed by Sanofi and Janssen, aimed to prevent ExPEC sepsis in older adults with a history of UTIs. Initially formulated as ExPEC10V, the vaccine included ten O-antigen (OAg) serotypes but was later revised to ExPEC9V after phase 1/2a trials revealed poor immunogenicity for O8 [104]. The vaccine advanced to a phase 3 trial (NCT04899336), enrolling nearly 20,000 adults over age 60 across multiple countries. However, in February 2025, an independent monitoring board determined that ExPEC9V was not sufficiently effective at preventing invasive ExPEC disease. Although no safety issues were reported, the lack of significant efficacy led the manufacturers to discontinue the program.

The other clinical-stage candidate, developed by Sequoia Pharmaceuticals, targets FimH, an *E. coli* adhesion protein essential for bladder epithelial cell attachment. Sera from FimH-vaccinated animals inhibited *E. coli* adhesion to human bladder cells in vitro, while immunization reduced bladder colonization by over 99% in a murine cystitis model, with vaccinated mice showing FimH-specific IgG in urine samples [105]. More recently, a recombinant FimH vaccine adjuvanted with liposomal QS21/MPLA demonstrated strong immunogenicity and protection in a cynomolgus macaque UTI model. Compared to placebo, vaccinated animals exhibited >200-fold reductions in bacteriuria by day 2 and >700-fold by day 7 post-infection, along with significantly lower urinary inflammatory biomarkers [106]. Following a successful phase 1 clinical trial in the U.S., the FimH vaccine is now advancing to phase 2 to further assess its efficacy in preventing ExPEC-associated UTIs [107].

MV140 (Uromune) is a sublingual vaccine developed to prevent recurrent UTIs. It contains inactivated whole cells from four bacterial species: *E. coli, Klebsiella pneumoniae, E. faecalis,* and *Proteus vulgaris* [108]. In a representative prospective study of 77 women with recurrent UTIs, 3-month sublingual vaccination with MV140 (Uromune) resulted in 78% (59/75) remaining UTI-free over a 12-month follow-up, despite all participants having experienced ≥3 UTIs in the prior year [109]. MV140 is currently in a pre-licensed phase 2/3 study and is available under special access programs in countries including Spain, Portugal, and the U.K. [108]. This vaccine shows promise as an non-antibiotic alternative for managing recurrent UTIs and associated healthcare expenditures [110].

Several innovative ExPEC vaccine candidates are in preclinical development. One candidate targets SinH, an autotransporter conserved across many ExPEC serotypes, which has shown potential for broad protection in mouse models [111]. Another strategy involves OMVs from *E. coli* coated onto 30 nm gold nanoparticles to improve stability and antigen-presenting cell uptake [112]. In murine models, these BM-AuNPs induced potent dendritic cell activation, high-avidity antibody responses, and a Th1/Th17-skewed immune profile, highlighting their potential for effective antibacterial vaccine development [112]. A live-attenuated *E. coli* K1 (∆*aroA*) vaccine also demonstrated robust immunogenicity in mice. Maternal vaccination produced bactericidal antibodies that were transferred to pups, providing protection against both K1 and non-K1 *E. coli* strains—suggesting its potential use in pregnant women at risk for preterm delivery to protect neonates from severe ExPEC infection [113].

While progress on ExPEC vaccines has been mixed, parallel efforts on enterotoxigenic *E. coli* (ETEC) vaccines remain active, with several formulations (e.g., ETVAX^®^ and ACE527) advancing through clinical trials in both endemic populations and travelers [114,115,116,117]. These programs, though distinct from ExPEC, highlight the broader momentum in *E. coli* vaccine development and may inform future strategies for preventing invasive ExPEC disease. Importantly, preliminary findings indicate that ETVAX and ACE527 are well tolerated, generate robust mucosal and systemic immune responses across age groups, and show encouraging protection signals in early studies, although definitive phase 3 efficacy data are still pending [114,115,116,117].

### 4.5. Salmonella spp.

*Salmonella enterica* serovar Typhi (*S*. Typhi), the causative agent of typhoid fever, poses a major public health threat, particularly across Asia and sub-Saharan Africa [14]. Once readily treatable with first-line antibiotics, typhoid fever has become increasingly difficult to manage due to the emergence of MDR and XDR strains of *S*. Typhi, driven in part by inappropriate antibiotic use and empirical therapy [118,119]. Currently, four typhoid conjugate vaccines (TCVs) are prequalified by the WHO: Typbar-TCV^®^ (Bharat Biotech, 2017), TYPHIBEV^®^ (Biological E, 2020), ZyVac-TCV (Zydus Lifesciences, 2024), and SKYTyphoid™ (SK Bioscience, 2024). Additional candidates such as Vi-DT (BioFarma) have received regulatory approval in Indonesia (marketed as Bio-TCV^®^) and are under review for WHO prequalification. TCVs, which conjugate Vi polysaccharide to carrier proteins such as tetanus toxoid or CRM197, are the preferred option for infants and young children due to their superior immunogenicity and durability [120,121]. A randomized trial in Nepal demonstrated 81.6% efficacy in children aged 9 months to 16 years, and pooled estimates across diverse settings range from 79–95% [120,122]. Although comprehensive cost-effectiveness data remain limited, modeling suggests substantial savings in healthcare and household expenditures following widespread TCV implementation [123].

Despite these advances, the global burden of *Salmonella* infections is not limited to *S*. Typhi. Non-typhoidal *Salmonella* (NTS) serotypes—including *S*. Paratyphi A, *S*. Typhimurium, and *S*. Enteritidis—also contribute significantly to global morbidity. *S*. Paratyphi A causes paratyphoid fever in endemic parts of Asia, while *S.* Typhimurium and *S*. Enteritidis are leading causes of invasive non-typhoidal salmonellosis (iNTS), particularly in immunocompromised populations in sub-Saharan Africa [124]. However, no licensed vaccines currently exist for these serotypes. A recent study introduced a quadrivalent *Salmonella* vaccine based on the Multiple Antigen Presenting System (MAPS) platform, targeting *S*. Typhimurium, *S*. Enteritidis, *S*. Typhi, and *S*. Paratyphi A [125]. This combinational MAPS vaccine induced broad immunity in preclinical models and holds promise as a unifying strategy against both typhoidal and non-typhoidal *Salmonella* disease.

Vaccine development for *S*. Paratyphi A has progressed slowly, in part due to limited commercial interest and the large sample sizes required for efficacy studies. The most advanced candidate is a conjugate vaccine linking *S*. Paratyphi A-specific O:2 antigen to tetanus toxoid (O:2-TT), which demonstrated safety and immunogenicity in Phase 1 and 2 trials in Vietnam but has not progressed since 2000 [126]. More recent approaches include a CRM197-conjugated O:2 vaccine evaluated in murine models [127] and an oral live-attenuated strain, CVD 1902, which completed a Phase 1 trial in the U.S. [128]. Notably, a controlled human infection model (CHIM) has now been established for CVD 1902, enabling a planned Phase 1/2 efficacy trial and potentially accelerating the development of additional candidates [129].

Several bivalent vaccine candidates combining *S*. Typhi and *S*. Paratyphi A antigens are also under investigation. Entervax, a live oral combination of Typhi ZH9 and an engineered ZH9PA strain expressing *S*. Paratyphi A LPS O:2 and H:a flagella, completed a Phase 1 trial (NCT04349553) [130]. A conjugate vaccine developed by the Serum Institute of India containing Vi-TT and O:2-DT has shown safety and immunogenicity in a Phase 1 trial [131]. Another bivalent Vi-CRM197+O:2-CRM197 formulation developed by GSK Vaccines for Global Health (GVGH) and Biological E has entered clinical testing, supported by strong preclinical data [132,133].

For iNTS pathogens *S.* Typhimurium and *S.* Enteritidis, which are major contributors to pediatric bloodstream infections in Africa, bivalent vaccines are under active investigation. One promising platform is the Generalized Modules for Membrane Antigens (GMMA), used to engineer STmGMMA from a modified *S*. Typhimurium strain. This candidate has demonstrated strong immunogenicity in murine studies and may be expanded to include *S*. Enteritidis [134]. Another approach is a bivalent glycoconjugate vaccine that combines O-antigens from both serotypes with CRM197 and CpG/aluminum hydroxide adjuvants. This formulation elicited robust antibody responses in mice [135] and is currently being evaluated in a Phase 1/2a clinical trial (NCT05480800), expected to conclude in late 2024. A parallel trial—the SALVO study—is also underway in the UK to assess iNTS-GMMA in an observer-participant blind placebo-controlled design [136].

### 4.6. Shigella spp.

Shigellosis, caused by the *Shigella* genus (*S. dysenteriae*, *S. flexneri*, *S. boydii* and *S. sonnei*) can result in severe diarrheal disease [137]. An estimated 165 million cases and 1 million deaths occur annually worldwide due to *Shigella*, with 50% of these in children under 5 years of age. Resistance to commonly prescribed antibiotics such as cephalosporins and fluoroquinolones is increasing [138]. Currently, there are no licensed *Shigella* vaccines, but several promising candidates are under development.

One of the most advanced *Shigella* vaccine candidates is ZF0901, a bivalent conjugate vaccine targeting *S. flexneri* 2a and *S. sonnei* [139,140]. It conjugates the O-antigen (OAg) of these serotypes to tetanus toxoid, making it one of the few vaccines designed to provide broad serotype coverage. In a phase 2 trial involving children aged 3 months to 5 years in China, ZF0901 was well-tolerated, demonstrated acceptable reactogenicity, and induced a strong immune response [140]; it has now advanced to a phase 3 trial [139]. Another promising candidate, altSonflex1-2-3, utilizes GMMA (Generalized Modules for Membrane Antigens) technology, which employs bacterial OMVs to deliver OAgs to the immune system [141]. This vaccine, incorporating GMMAs from *S. sonnei* and *S. flexneri* 1b, 2a, and 3a, has shown strong immunogenicity and safety in preclinical studies [142]. The GMMA-based *S. sonnei* vaccine 1790GAHB has been evaluated in multiple trials. A phase 2b controlled human infection model (CHIM) study found that although the vaccine was safe and immunogenic, it did not confer clinical protection against shigellosis; however, higher pre-challenge anti-LPS IgG levels were associated with protection, suggesting potential correlates of immunity [143]. In a phase 2a study conducted in Kenya, 1790GAHB elicited strong anti-GMMA protein IgG responses, though these did not correlate with clinical protection [144]. Additional immunological profiling of 1790GAHB has demonstrated induction of α4β7^+^ LPS-specific B cells, suggesting the potential for both systemic and mucosal responses, although definitive correlates of protection remain elusive.

The S4V vaccine is a quadrivalent bioconjugate targeting *S. sonnei* and *S. flexneri* 2a, 3a, and 6 [145]. It conjugates the OAgs of these serotypes to the carrier protein EPA and is currently being evaluated in a phase 1/2 trial in Kenya to assess immunogenicity, safety, and optimal dosage in infants [145]. Beyond these candidates, other *Shigella* vaccine approaches include live-attenuated strains [146], and subunit vaccines, including a multiepitope fusion antigen (MEFA) vaccinology platform, where epitope- and structure-based polyvalent proteins are constructed to induce cross-protective antibodies against a variety of *Shigella* or enterotoxigenic *E. coli* strains [147]. More recently, a quadrivalent *Shigella* MAPS vaccine targeting *S. flexneri* 2a, 3a, 6, and *S. sonnei* was shown to induce broad antibody responses in preclinical models and offers a modular platform for expanding serotype coverage [148].

### 4.7. Klebsiella pneumoniae

*Klebsiella pneumoniae* is a leading cause of hospital-acquired infections including pneumonia, UTI, and bloodstream infections, with expanding multidrug-resistance [149] and increasing prominence as a cause of neonatal sepsis and pneumonia in low- and middle-income countries [150]. The rising prevalence of hypervirulent *K. pneumoniae* ST23 strains carrying carbapenemase genes is particularly concerning, as these strains are not only highly resistant but also cause severe invasive infections in both healthy and immunocompromised individuals [151]. A major challenge complicating *K. pneumoniae* vaccine design is the pathogen’s extensive capsule variability, with limited cross-protection—at least 77 serologically distinct *Klebsiella* capsular (K) types have been identified, while genotyping has revealed 147 different K loci [152]. In the late 1980s, the Swiss Serum and Vaccine Institute developed Klebvax^®^, a multivalent unconjugated capsular polysaccharide vaccine including 24 distinct *K. pneumoniae* capsular serotypes. Despite promising preclinical results [153], clinical trials revealed that the vaccine induced only T-cell-independent immunity, failing to elicit immunological memory, high-affinity antibody production, or disease protection. Consequently, the vaccine was not pursued further.

Conjugation would be expected to improve polysaccharide immunogenicity [154], though it may not resolve the capsule variability conundrum. In contrast to the capsular polysaccharide, *K. pneumoniae* has only 11 known O-antigen serogroups, with four (O1, O2, O3, and O5) representing over 80% of clinical isolates worldwide [155]. Kleb4v (GSK and LimmaTech) is a quadrivalent bioconjugate vaccine targeting the O-polysaccharide subtypes O1, O2a, O2afg, and O3b [156]. This candidate underwent a phase 1/2 study (NCT04959344) for safety and immunogenicity in individuals aged 55–70, with and without the adjuvant AS03, but was terminated in 2022 with limited information on outcomes. In preclinical studies, a glycoconjugate vaccine combining *K. pneumoniae* O1, O2, O3, O5 antigens with *Pseudomonas aeruginosa* flagellin proteins enhanced anti-polysaccharide immune responses and generated strong antibody titers in rabbits [157]. Passive transfer of vaccine-induced antisera reduced bacterial burden and protected mice from fatal *K. pneumoniae* infection, supporting its potential as a broader-coverage vaccine candidate [157]. Similarly, a heptavalent *K. pneumoniae* bioconjugate vaccine targeting seven predominant O-antigen subtypes elicited antibodies with varying binding and complement-mediated killing activity against multiple strains, although increased capsule production in a subset reduced antibody binding and killing efficacy—highlighting a potential challenge in achieving broad coverage [158].

Several innovative preclinical approaches have also emerged. A *K. pneumoniae* bacterial ghost (BG) vaccine was generated using an optimized holin–endolysin lysis system, producing frameless bacterial envelopes [159]. BG immunization in mice elicited strong humoral and cellular immune responses and protected against infection by a hypervirulent *K. pneumoniae* O1:K2 strain [159]. A lipid nanoparticle mRNA vaccine targeting YidR, a highly conserved protein implicated in *K. pneumoniae* hyperadherence, induced a strong Th1-biased immune response, reduced bacterial load, and increased survival in a murine lung infection model [160]. Finally, outer membrane protein A (rOmpA) was encapsulated in silk fibroin/sodium alginate nanoparticles, demonstrating high encapsulation efficiency and sustained antigen release. This nanoparticle formulation elicited strong systemic and mucosal immune responses, enhanced Th1-polarized immunity, and protected against bacterial proliferation and lung inflammation in a murine pneumonia model [161].

### 4.8. Neisseria gonorrhoeae

*Neisseria gonorrhoeae* is a common sexually transmitted pathogen that can lead to a range of clinical syndromes, including urethritis, cervicitis, and disseminated gonococcal infection. In women, untreated infections can result in pelvic inflammatory disease, increasing the risk of infertility. The WHO has identified gonorrhea as a growing AMR threat [162], with rising reports of MDR strains, particularly those linked to international travel from Southeast Asia [163]. Developing a gonococcal vaccine is crucial, but challenges such as the antigenic diversity of *N. gonorrhoeae* surface proteins and the lack of a reliable animal model have slowed progress [164].

Natural infection by *N. gonorrhoeae* does not confer immunity, and no approved vaccine is currently available, although some evidence suggests cross-protection from existing meningococcal vaccines could be meaningful. The *N. meningitidis* 4CMenB vaccine (Bexsero, GSK) contains recombinant protein antigens and OMVs, with NHBA and OMV-derived antigens likely contributing to cross-protection against *N. gonorrhoeae* [165]. Numerous trials of 4CMenB across several countries—mostly enrolling young adults aged 15–30—were recently analyzed in a systematic manner for protection against any gonococcal infection, with pooled efficacy estimates ranging from 22% to 46% [166]. A long-term study in New Zealand found that the meningococcal OMV-based vaccine MeNZB offered 31% protection against gonorrhea [167]. In Cuba, following a mass vaccination campaign with the *N. meningitidis* serogroup B OMV vaccine VA-MENGOC-BC and its inclusion in the national immunization program, gonorrhea rates declined sharply—contrasting with trends in other sexually transmitted diseases; additional reductions in unvaccinated age groups suggested some degree of herd immunity [168].

Another platform applied to gonococcal OMV vaccine development is GMMA [169], in which the bacterium is engineered to yield an over-vesiculating phenotype, reducing development costs. NgG, a GMMA *N. gonorrhoeae* vaccine from GSK, is undergoing a phase 1/2 trial to assess safety and efficacy in healthy adults aged 18 to 50 in multiple countries. The trial is expected to conclude in 2025 (Clinical Trial ID: NCT05630859) and has received FDA fast-track designation to expedite development [170]. Additionally, an optimized OMV vaccine was recently developed by deleting the immunosuppressive protein RmpM and replacing gonococcal PorB with meningococcal PorB, which has adjuvant properties. In a mouse model, this altered OMV formulation elicited stronger and more diverse IgG antibody responses, increased IFN-γ production, and induced a Th1-skewed immune response.

Recent studies have explored nanotechnology-based vaccine platforms for *N. gonorrhoeae*, employing inactivated whole-cell gonococcal microparticles (Gc-MPs) encapsulated in biodegradable matrices and delivered via dissolvable microneedle (MN) patches for transdermal immunization. These nanoparticle formulations, often co-delivered with adjuvant-loaded microparticles such as Alum MP or AddaVax™ MP, have demonstrated robust induction of antigen-specific humoral and cellular immune responses, including mucosal IgA and serum bactericidal antibodies [171,172].

### 4.9. Pseudomonas aeruginosa

*Pseudomonas aeruginosa* remains a leading cause of healthcare-associated infections, including ventilator-associated pneumonia, bloodstream infections, UTIs, and chronic respiratory infections in individuals with cystic fibrosis [173]. Its pathogenicity is driven by a broad range of virulence factors, including type III secretion system effectors (ExoS, ExoT, ExoU), elastases, pyocyanin, and robust biofilm formation that enhances immune evasion and persistence [174]. The global rise in MDR and XDR *P. aeruginosa*, particularly involving carbapenemases such as VIM and NDM variants, has led the WHO to reclassify it as a critical priority pathogen for antibiotic development [175]. About 45.9% of *P. aeruginosa* cases are MDR, and 9.5% are XDR, with high resistance to gentamicin and cephalosporins [176].

Despite decades of intensive research, no vaccine against *P. aeruginosa* has been approved for clinical use. While numerous candidates have demonstrated immunogenicity, challenges such as limited efficacy in preventing chronic pulmonary infections, safety concerns, and the pathogen’s adaptability and virulence have impeded progress [177]. Only a small number of candidates have reached clinical trials, with most programs either terminated, stalled, or unpublished following early results. For example, IC43 (Valneva SE/IntercellAG), a recombinant vaccine of outer membrane proteins OprF/Opr1, completed phase II trials in ventilated ICU patients. Although the vaccine achieved high immunogenicity, it did not reduce overall mortality compared to placebo [178]. Medimmune’s Aerogen^®^, a detoxified LPS conjugate vaccine studied in cystic fibrosis patients, showed promise in early studies but has not progressed further. Successful *P. aeruginosa* vaccine development will likely require strategies that elicit both strong mucosal and systemic immunity, with a particular emphasis on generating protective IgA responses and Th17-mediated immunity at respiratory sites [177].

Recently, immunization with CbpD, a lytic polysaccharide monooxygenase implicated in *P. aeruginosa* virulence during pneumonia, provided robust protection in mice through antibody-mediated neutrophil opsonophagocytosis [179]. However, this and prior efforts have targeted single antigens such as OprF and OprI, but their inability to provide broad or durable protection has prompted the search for multivalent approaches [180]. A semisynthetic oligomannuronic acid–based glycoconjugate vaccine provided protection against both mucoid and non-mucoid *P. aeruginosa* in preclinical models, underscoring the potential of conserved polysaccharide antigens as cross-protective targets [181]. In parallel, reverse vaccinology–guided multi-epitope vaccine design has identified conserved CTL, HTL, and B-cell epitopes predicted to generate robust humoral and cellular immunity across diverse strains [182]. Another study used X-ray-inactivated *P. aeruginosa* (XPa), which protected mice in a pneumonia model and suggested a path forward for whole-cell inactivation strategies [183]. A dual-antigen recombinant vaccine combining PcrV and AmpC elicited a Th17-biased immune response and conferred protection in preclinical models, although its safety in immunocompromised hosts remains under investigation [184].

More recently, mRNA vaccine platforms—inspired from successful COVID-19 vaccines—have been applied to *P. aeruginosa*. One study using lipid nanoparticle-encapsulated mRNA encoding PcrV or a fusion of OprF-I demonstrated robust humoral and cellular immune responses, with protection against both systemic and burn wound infections in mice [185]. Nanovaccine strategies have also emerged, including a formulation employing hybrid membrane vesicles (AuNP@HMV@SPs) composed of bacterial OMVs and macrophage membranes coated onto gold nanoparticles. This formulation induced strong immune responses and conferred complete protection in models of septicemia and pneumonia [186]. Likewise, a STING-adjuvanted outer-membrane vesicle (OMV) *P. aeruginosa* vaccine elicited durable mucosal and systemic responses with protective efficacy in murine pneumonia and sepsis models [187].

### 4.10. Acinetobacter baumannii

*Acinetobacter baumannii* is a Gram-negative, opportunistic pathogen and a leading cause of hospital-acquired infections, including ventilator-associated pneumonia (VAP), bloodstream infections, and wound infections, particularly in ICUs and among immunocompromised individuals [188]. The prevalence of MDR strains is alarmingly high. In hospital-acquired pneumonia (HAP) and VAP, MDR rates range from 56.5% to 100% in some regions, with global estimates indicating that nearly 80% of clinical isolates exhibit resistance to at least three major antibiotic classes [189]. Vaccine development against *A. baumannii* remains challenging due to its genetic diversity, capacity for biofilm formation, and the lack of well-defined protective antigens.

Preclinical vaccine research against *A. baumannii* has explored a wide range of strategies to counter this MDR pathogen. Reverse vaccinology has identified novel antigen candidates; for example, a study analyzing 14 *A. baumannii* genomes pinpointed 13 potential vaccine targets, of which three antigens demonstrated high immunogenicity and conferred 60% protection in a pneumonia mouse model [190]. Pan-genomics and subtractive proteomics have supported the design of chimeric vaccines incorporating B-cell and T-cell epitopes from proteins such as APN, AdeK, and AdeI, which showed promising interactions with host immune receptors [191]. Multi-epitope subunit vaccines have also been investigated; one such construct, combining linear B-cell, cytotoxic T lymphocyte (CTL), and helper T lymphocyte (HTL) epitopes from LPS assembly proteins LptE and LptD, elicited robust neutralizing antibody responses [192]. In parallel, a novel mRNA-based multi-epitope vaccine demonstrated potent immune responses and minimal side effects in preclinical models [193].

Glycoconjugate vaccines represent another promising approach. One study engineered an O-linked glycosylation system in *A. baumannii* to produce a conjugate vaccine that elicited strong Th1 and Th2 immune responses, reduced bacterial loads, and protected against lethal sepsis in murine models [194]. A separate pentavalent formulation, combining recombinant proteins Wza and YiaD with capsular polysaccharides from three clinical isolates, achieved 100% survival in mice challenged with diverse *A. baumannii* strains [195]. Additional innovations include a fusion protein vaccine in which the outer membrane protein Omp22 is embedded into the hypervariable domain of flagellin (FliC-Omp22), enhancing immunogenicity and protecting mice from lethal infection [196]. Whole-cell vaccines inactivated by irradiation, derived from both planktonic and biofilm-like cultures, have also demonstrated protective efficacy in mice [197]. A promising preclinical candidate, Ab-NP, uses gold nanoparticles coated with OMVs derived from the hypervirulent *A. baumannii* strain Lac-4. This formulation conferred durable protection against lethal sepsis and pneumonia for up to six months without the need for additional boosters [198].

### 4.11. Clostridiodes difficile

*Clostridiodes difficile* causes symptomatic infections in approximately 500,000 people in the U.S. each year, including severe and sometimes life-threatening cases of toxin-mediated pseudomembranous colitis [199]. Disease development is often triggered by antibiotic use, and many *C. difficile* strains exhibit AMR, including resistance to fluoroquinolones, clindamycin, and erythromycin, with a high prevalence of MDR variants [200]. A toxoid vaccine, PF-06425090 (Pfizer), targeting the high molecular weight toxins A (TcdA) and B (TcdB), showed promise in early trials but did not meet the primary endpoint for preventing disease in a phase 3 trial in adults over age 50. However, the vaccine achieved several secondary efficacy endpoints (e.g., shortened disease duration) and demonstrated a favorable safety profile, suggesting potential for further development [201,202]. Another candidate, VLA84 (Valneva, Austria)—a fusion protein vaccine containing key epitopes from TcdA and TcdB—showed safety and immunogenicity in a phase 2 study, though its phase 3 trial is currently on hold [203]. GSK2904545A (GSK), likewise a *C. difficile* TcdA and TcdB fusion protein vaccine, completed a phase 1 study in 2022, evaluating safety and immunogenicity across two age groups [202].

There is ongoing debate about whether vaccines against *C. difficile* should focus solely on neutralizing toxins or also target surface proteins involved in colonization and spore formation. A recent multivalent mRNA vaccine [204] embraced a dual approach, encoding both the toxins TcdA and TcdB and Pro-Pro endopeptidase 1 (PPEP-1), a key virulence factor involved in motility and adhesion. In rodent models, this mRNA vaccine elicited a more potent immune response than traditional alum-adjuvanted recombinant protein vaccines, with a single dose inducing robust anti-toxin immunity and two doses generating PPEP-1-specific antibodies and T-cell responses [204]. Notably, mice vaccinated with the mRNA formulation exhibited 100% survival when challenged with five times the lethal toxin dose—outperforming protein-based vaccines, which provided limited or no protection [204].

## 5. Addressing AMR Through Vaccines: Strategic Imperatives and Future Directions

Antimicrobial resistance (AMR) represents a critical global health crisis, threatening the efficacy of current treatments and burdening healthcare systems—particularly in LMICs. While much of the focus has been on antibiotic stewardship and novel drug development, vaccines offer a powerful yet underutilized tool to prevent infections, reduce antibiotic consumption, and limit the selection and spread of resistant pathogens. Compared to antibiotics, vaccines are inherently less prone to resistance development, can induce herd immunity, and reduce reliance on empirical antibiotic therapy—thereby interrupting AMR evolution at its source (Figure 1).

Recent advances in vaccine technology—including bioconjugates, reverse vaccinology, generalized modules for membrane antigens (GMMAs), and nanoparticles—have introduced innovative strategies to improve vaccine efficacy. For instance, nanoparticles facilitate targeted delivery and potentiate antigen-presenting cell responses, thereby enhancing immunogenicity. Despite these promising developments, progress in vaccines targeting priority AMR pathogens remains limited. Development is constrained by lengthy timelines—often requiring 10–18 years—and high costs, posing a risky investment for pharmaceutical developers [205]. Additionally, lack of commercial incentives and health economic models that undervalue long-term and population-wide benefits, such as herd immunity or prevention of secondary infections, further discourage investment [206,207].

Moreover, evolutionary dynamics like serotype replacement can undermine vaccine impact, as illustrated by the rise in *S. pneumoniae* serotype 19A after PCV7 introduction and similar shifts following PCV13 rollout [208,209]. Nonetheless, the existence of licensed vaccines targeting AMR-relevant pathogens—including pneumococcus, *S.* Typhi, and *M. tuberculosis*—demonstrates the feasibility and transformative potential of vaccination as an AMR control measure. Pneumococcal conjugate vaccines (PCVs) have achieved substantial reductions in invasive pneumococcal disease (IPD), preventing 90% of IPD in healthy children [210,211] and 56% in adults [212].

Economic modeling further supports the integration of vaccines in AMR mitigation strategies. A recent WHO report estimates that vaccines targeting 23 AMR pathogens could cut global antibiotic use by 22%, equivalent to 2.5 billion defined daily doses annually [213]. Another study suggested that vaccines already in use—against Hib, pneumococcus, and *Salmonella* Typhi—could prevent approximately 106,000 AMR-associated deaths per year. This figure could rise six-fold if new vaccines against Mtb and *Klebsiella pneumoniae* are successfully developed and globally deployed [214]. In addition to lives saved, substantial cost savings are possible through reduced need for last-line antibiotics and curbed transmission of resistance organisms. For example, economic modeling in China—where antibiotic use is high and PCV coverage remains low—estimated that expanding PCV uptake to 99% could reduce AMR-related healthcare costs by USD 371-586 million, depending on the speed of the rollout [215]. These findings reinforce the role of vaccines as critical tools for reducing AMR-related clinical and economic burdens worldwide.

Despite this promise, stark global disparities in vaccine access persist. As of 2022, nearly 14 million children remained unvaccinated—most residing in LMICs where AMR burdens are greatest [216]. Encouragingly, international collaborations such as Gavi, the Vaccine Alliance, WHO’s Global Action Plan on AMR, International Vaccine Institute (IVI), and the Coalition for Epidemic Preparedness Innovations (CEPI) are directing investments toward bacterial vaccine development [217,218,219]. Sustained support and infrastructure development will be essential to ensure equitable access to these life-saving tools.

## 6. Conclusions

The decline in antimicrobial effectiveness coincides with significant advancements in vaccine sciences, offering new possibilities for developing AMR-targeted vaccines. Novel technologies—particularly mRNA platforms and structure-guided antigen design—offer unprecedented opportunities to generate AMR-targeted vaccines tailored to specific pathogens. The future of AMR prevention lies in strategically integrating these vaccine innovations with robust antimicrobial stewardship, surveillance, and infection control measures. This comprehensive, systems-level approach—grounded in innovation, global collaboration, and equitable implementation—will be critical to overcoming current barriers and achieving meaningful reductions in AMR prevalence worldwide.
